# Functional Activation of Osteoclast Commitment in Chronic Lymphocytic Leukaemia: a Possible Role for RANK/RANKL Pathway

**DOI:** 10.1038/s41598-017-12761-1

**Published:** 2017-10-26

**Authors:** Cecilia Marini, Silvia Bruno, Francesco Fiz, Cristina Campi, Roberta Piva, Giovanna Cutrona, Serena Matis, Alberto Nieri, Maurizio Miglino, Adalberto Ibatici, Anna Maria Orengo, Anna Maria Massone, Carlo Emanuele Neumaier, Daniela de Totero, Paolo Giannoni, Matteo Bauckneht, Michele Pennone, Claudya Tenca, Elena Gugiatti, Alessandro Bellini, Anna Borra, Elisabetta Tedone, Hülya Efetürk, Francesca Rosa, Laura Emionite, Michele Cilli, Davide Bagnara, Valerio Brucato, Paolo Bruzzi, Michele Piana, Franco Fais, Gianmario Sambuceti

**Affiliations:** 1 CNR Institute of Bioimages and Molecular Physiology, Milan, Italy; 2 Nuclear Medicine IRCCS AOU San Martino-IST, Genoa, Italy; 3 Nuclear Medicine, Department of Health Science, University of Genova, Genoa, Italy; 4 Department of Experimental Medicine, University of Genoa, Genoa, Italy; 5 Nuclear Medicine Unit, Department of Radiology, Uni-Klinikum Tuebingen, Germany; 6 SPIN Institute, CNR, Genoa, Italy; 7 Molecular Pathology, IRCCS AOU San Martino-IST, Genoa, Italy; 8 Department of Internal Medicine, University of Genoa, Genoa, Italy; 9 Haematology Clinic, IRCCS AOU San Martino-IST, Genoa, Italy; 10 Radiology Unit, IRCCS AOU San Martino-IST, Genoa, Italy; 11 Transfer Gene Laboratory, IRCCS AOU San Martino-IST, Genoa, Italy; 12 Stem Cell Laboratory, Department of Experimental Medicine, University of Genova, Genoa, Italy; 13 Animal Facility, IRCCS AOU San Martino-IST, Genoa, Italy; 14 Karches Center for Oncology Research, Feinstein Institute for Medical Research, Northwell Health, Manhasset, NY USA; 15 Department of Civil, Environmental, Aerospace, Materials Engineering, Aten Center, CHAB pole, University of Palermo, Viale delle Scienze, 6, Palermo, 90128 Italy; 16 Epidemiology and Clinical trial Service, IRCCS AOU San Martino-IST, Genoa, Italy; 17 Department of Mathematics (DIMA), University of Genoa, Genoa, Italy

## Abstract

Skeletal erosion has been found to represent an independent prognostic indicator in patients with advanced stages of chronic lymphocytic leukaemia (CLL). Whether this phenomenon also occurs in early CLL phases and its underlying mechanisms have yet to be fully elucidated. In this study, we prospectively enrolled 36 consecutive treatment-naïve patients to analyse skeletal structure and bone marrow distribution using a computational approach to PET/CT images. This evaluation was combined with the analysis of RANK/RANKL loop activation in the leukemic clone, given recent reports on its role in CLL progression. Bone erosion was particularly evident in long bone shafts, progressively increased from Binet stage A to Binet stage C, and was correlated with both local expansion of metabolically active bone marrow documented by FDG uptake and with the number of RANKL + cells present in the circulating blood. In immune-deficient NOD/Shi-scid, γcnull (NSG) mice, administration of CLL cells caused an appreciable compact bone erosion that was prevented by Denosumab. CLL cell proliferation *in vitro* correlated with RANK expression and was impaired by Denosumab-mediated disruption of the RANK/RANKL loop. This study suggests an interaction between CLL cells and stromal elements able to simultaneously impair bone structure and increase proliferating potential of leukemic clone.

## Introduction

B-cell chronic lymphocytic leukaemia (CLL) is the most frequent leukaemia in western countries^1,2^. It mainly occurs in the elderly, with 85–90% of patients being diagnosed after the age of 50^3^. Almost one third of CLL patients do not require any treatment during the entire disease course^4,5^. However, in the majority of patients CLL aggressiveness increases at a variable time after diagnosis, because of the combination of antiapoptotic mechanisms^5^ and enhanced proliferating activity^6–10^_._ Factors triggering this progression are still not fully clarified and no treatment has shown capability to halt disease progression in the asymptomatic phase^11,12^. However, there is accumulating evidence that the TNF superfamily member Receptor Activator of Nuclear Factor Kappa-B Ligand (RANKL) and its receptor RANK do play a role in CLL clone upkeep and in the progression of other B-cell related hematologic malignancies^13–18^. This pathway is indeed crucial in the immune system’s physiological development, as mice lacking RANK show absent lymph node development and impaired B-Cell differentiation^19^. In CLL, the recognized abundance of surface RANKL is often paralleled by an aberrant RANK expression^14–16^. On one side this phenomenon leads to an upregulation of interleukin-8 expression/release improving clone survival and expansion^14^. On the other hand, the capability of this same axis to promote osteoclast commitment in circulating monocytes enhances bone reabsorption possibly leading to the release of a variety of growth factors into the bone microenvironment as recognized in multiple myeloma and bone metastases^15,20,21^.

Using a dedicated software, capable of quantifying volumes of compact (CBV) and trabecular bone (IBV) from X-ray CT slices^22–24^, we already documented a selective erosion of compact bone, which managed to predict patients’ disease-specific survival, providing prognostic information independent from the commonly employed biomarkers^23^. Similarly, Lagenberg *et al*. reported the occurrence of pathological fractures in a patient with chronic lymphatic leukaemia without disease progression^25^.

The present study was designed to verify whether this alteration characterizes CLL per se or only occurs in its advanced stages and involves RANK/RANKL loop. Accordingly, we prospectively recruited a cohort of treatment-naïve CLL patients to simultaneously evaluate global skeletal volume (SV) and structure, active bone marrow (RBM) distribution and metabolism as well as clonal activation by RANK/RANKL pathway. The data obtained represents evidence about the role of this paracrine/autocrine loop in skeletal derangement and proliferating activity of the CLL neoplastic clone.

## Results

### Clinical staging and demographic characteristics

According to the clinical characteristics reported in Table 1, the study population was divided into 16, 12 and 8 patients as Binet A, B and C stages, respectively. Age, sex distribution and time elapsed from diagnosis to imaging were not significantly different in the three risk classes. Similarly, molecular indexes of disease aggressiveness were not significantly associated with Binet stages. In fact, >30% CD38^+^ cell prevalence was superimposable (50%) in all three classes while IgHV mutation in <2% clonal cells classified as high risk 25%, 42% and 63% subjects in Binet A, B and C stages, respectively (p < 0.01) (Table 1). Binet C stage was obviously associated with significantly lower levels of hemoglobin as well as with higher counts of both circulating leucocytes and CD19^+^ cells.

**Table 1:** Clinical Characteristics of the Pateints’ Population.

Group	Number	Gender F/M	AGE (Years)	HEIGHT (cm)	WEIGHT (Kg)	Disease Duration (Months)	Hb (g/L)	WBC (10E9/L)	PLT (10E9/L)	LYMPHOCYTES (RELATIVE %)	%CLONE	IgHV mutation in >2% cells	CD38%
All	36	11/25	70 ± 12	168 ± 8	71 ± 9	34 ± 39	126 ± 22	62,58 ± 55,6	180 ± 56,5	73 ± 20,4	63,24 ± 24,9	22 (61%)	34 ± 22
Binet A	16	6/10	71 ± 13	166 ± 7	66 ± 7	35 ± 33	136,2 ± 14,9	43,7 ± 39	193,3 ± 42,8	68,4 ± 19,4	55,67 ± 21	12 (75%)	30 ± 18
Binet B	12	4/8	67 ± 12	166 ± 9	77 ± 9	39 ± 46	129,6 ± 14	61,1 ± 46,2	77,2 ± 38,9	77,2 ± 14,7	61 ± 30	7 (58%)	38 ± 24
Binet C	8	1/7	73 ± 9	172 ± 7	72 ± 10	23 ± 43	105,5 ± 26,5	102,4 ± 78,1	154 ± 86,5	75,9 ± 28,4	81,7 ± 12	3 (37%)	36 ± 25

PET/CT scans did not show lesions with elevated glucose metabolism in any patient. Lymph nodal enlargement in > 2 stations was observed in 30/36 patients including 10/16 Binet A subjects (62%). However, these findings were not considered to modify clinical staging, according to the published guidelines^26^. At subsequent follow up (duration 14–27 months) no patient developed further neoplasia or chronic inflammatory conditions.

### Skeletal erosion and bone marrow glucose metabolism

**Figure 1:**
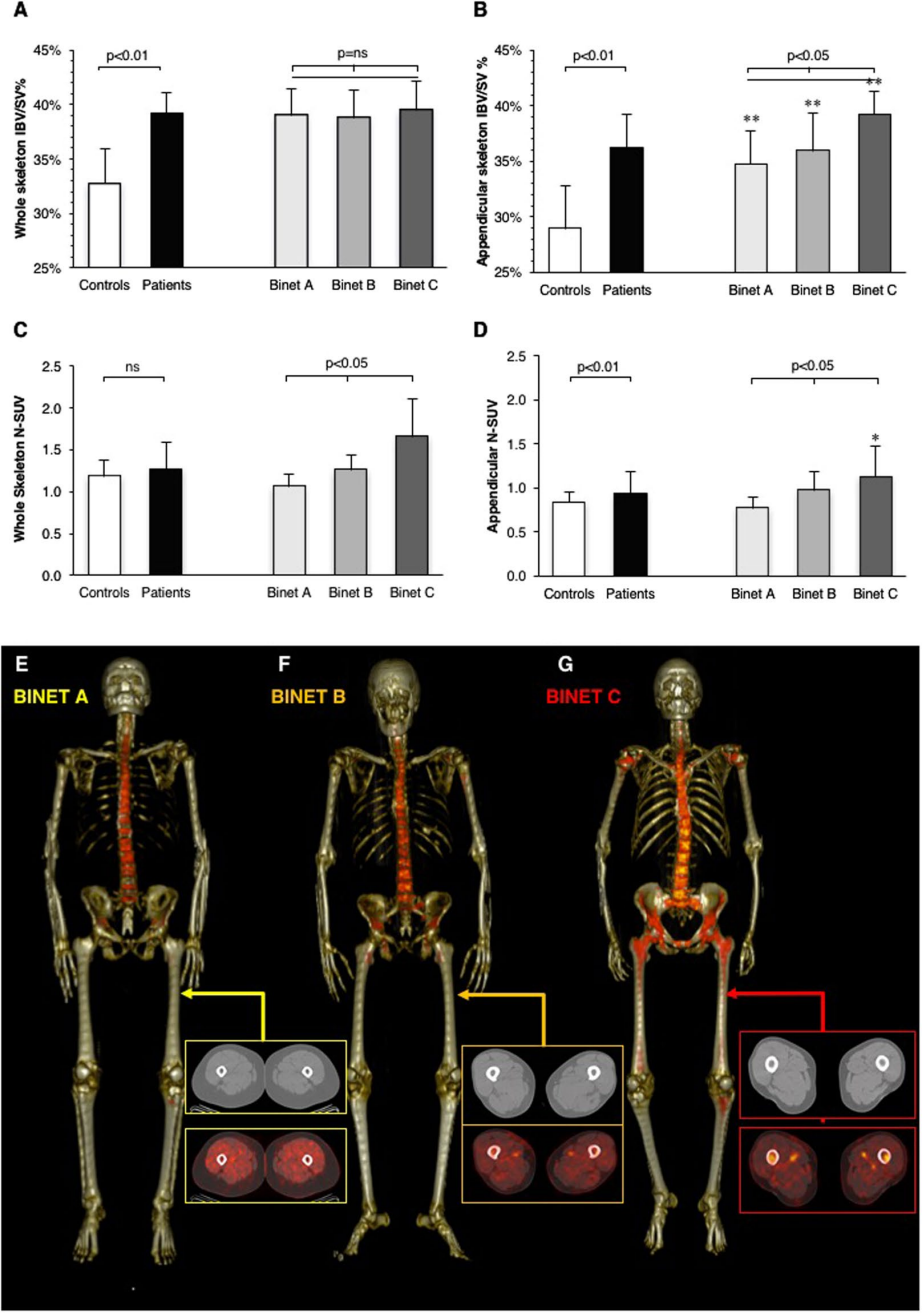
%IBV/SV ratio in control subjects (white) overall CLL patients (black). This latter group is subdivided according to Binet A (pale grey), B (grey) and C (dark grey). CLL was associated with a significant bone erosion (**p < 0.01 vs controls) that was independent from Binet stage when the whole skeleton was analyzed (panel A), while it was related to clinical disease stage in the appendicular districts (panel B). Panel C shows whole body RBM glucose consumption in CLL patients and control subjects. In appendicular bones (Panel D), this same pattern was associated with a significant progressive trend in N-SUV (p < 0.05) between the different Binet stages and became higher than control value in Binet C patients (*p < 0.05). Panels E-F-G: Examples of RBM distributions in the three Binet stages, respectively. The expansion of RBM in proximal femur shafts is evident only in Binet C patient (red arrows).

Volume of the entire skeleton was comparable in patients and in control subjects (66 ± 11vs 66 ± 9 ml/Kg IBW, respectively, p = ns). Nevertheless, CLL was associated with a significant erosion of compact bone paralleled by an expansion of IBV. In fact, IBV/SV ratio of the whole skeleton was higher in patients than in control subjects (39.1 ± 2% vs 32.7% ± 3.2%, respectively, p < 0.001, Fig. 1A). In the axial segments, this phenomenon was barely appreciable (51.1 ± 2.5% vs 47 ± 5.9%, respectively, p = 0.08). By contrast, it was particularly manifest in the appendicular bones (36.2% ± 2.1% in patients vs 28.9% ± 3.9% in controls, respectively, p < 0.001, Fig. 1B). In fact, shaft cortical thinning occurred in all Binet classes and showed a progressive increase from stage A to stage C (trend analysis p = 0.011, Fig. 1B). Finally, IBV/SV ratio was virtually independent from clinical features such as age, time elapsed from disease diagnosis or IgHV mutation (Suppl. Figure 1).

Average FDG uptake in RBM (defined by normalized standardized value N-SUV) was similar in CLL patients and control subjects (1.23 ± 0.3 vs. 1.18 ± 0.2, respectively, p = ns, Fig. 1C). However, the influence of CLL on BM metabolic activation was suggested by the progressive increase in FDG uptake from stage A to stage C that occurred in the whole skeleton and in appendicular segments (trend analysis p < 0.05) (Fig. 1C,D). This pattern was particularly evident in long bone shafts in which RBM N-SUV progressively increased throughout Binet stages to become significantly higher with respect to controls in stage C (Fig. 1D,E–G).

The patient-by-patient analysis did not document any relationship between RBM glucose consumption and bone erosion in the whole skeleton or in axial districts (Suppl. Figure 2). By contrast, there was a fair correlation (r = 0.57, p < 0.01) between IBV/SV ratio and metabolically active hematopoietic tissue in the appendicular segments (Suppl. Figure 2).

### CLL cells capability to impair bone structure in xenograft model

**Figure 2:**
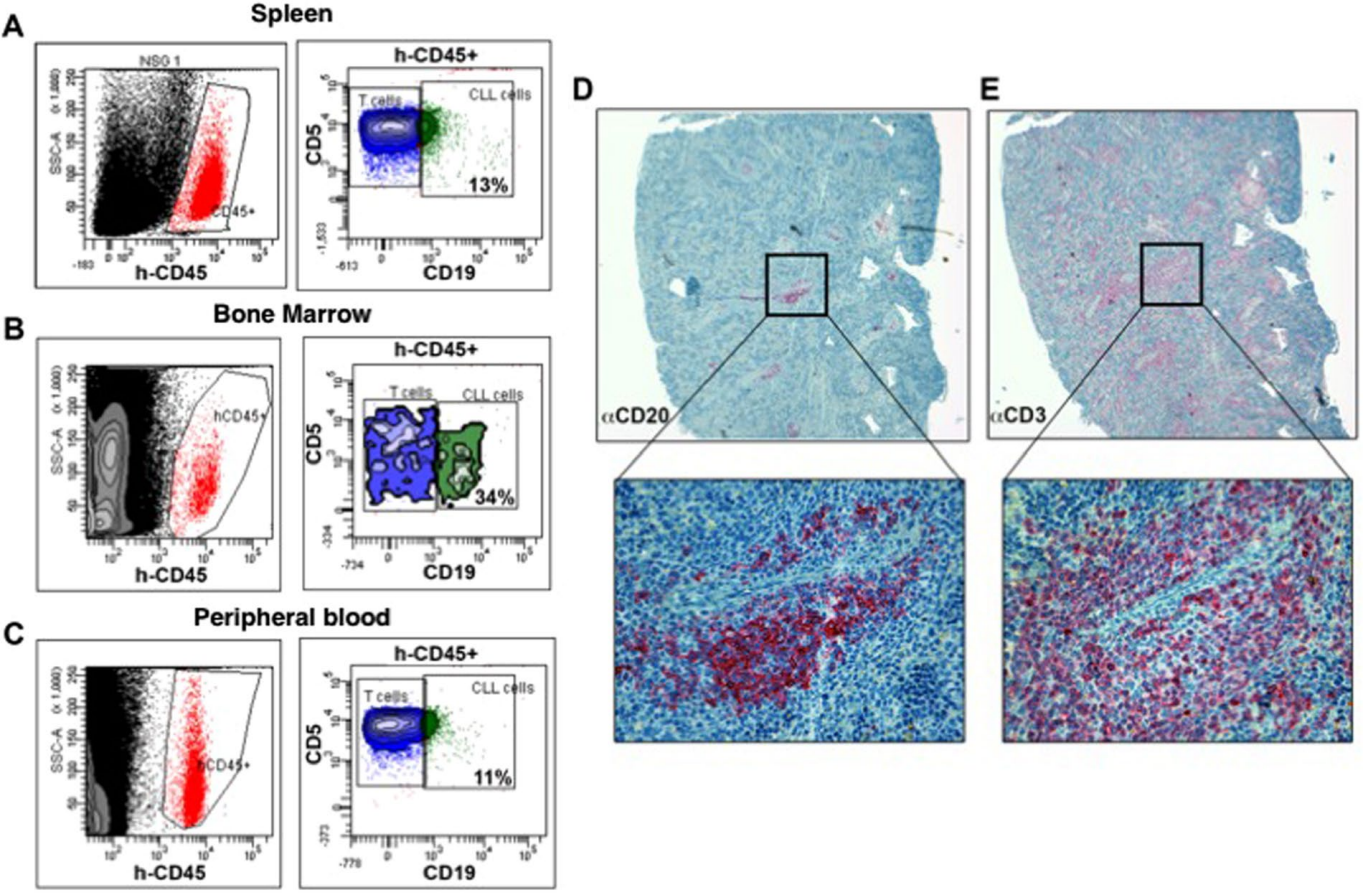
Panels A–C report the detection of the presence of leukemic cells engrafted in the spleen (**A**), BM (**B**) and peripheral blood (PBMC, **C**) as h-CD45/CD19/CD5-triple positive cells by flow cytometry in a representative mouse injected intravenously with the PBMC cells from a CLL patient. Panels D and E show immunohistochemical (IHC) analysis in paraffin embedded tissue slice sections from the same spleen. Tissue section is analysed for the presence of the typical neoplastic foci positive for CD20 B cell marker (**D**) surrounded by autologous CD3^+^ T cells (**E**) (magnification 40x upper panels and 400x lower panels).

One of the possible mechanisms underlying the association between clinical CLL stage and bone erosion is represented by the activation of RANK/RANKL pathway. To verify this hypothesis, 20 NOD-SCID IL-2R γ-chain null (NSG) female mice were intravenously inoculated with 8 × 10^7^ mononucleated cells collected from peripheral blood of two CLL patients (n = 10 each). Prior to cell administration, a high-resolution CT (CT1) documented that femur IBV/SV was similar in these animals with respect to the corresponding value in 10 control mice (data not shown). Four weeks after inoculation, successful engraftment was confirmed by flow cytometry of peripheral blood and a second high-resolution CT (CT2) was performed in all groups. Thereafter, CLL mice were divided into two subgroups to be treated with Denosumab (5 mg/Kg once a week for three weeks, n = 10) or with saline (n = 10) for three weeks before a further CT scan (CT3). Finally, all mice were sacrificed and cell suspensions from the spleen, bone marrow (BM), and peripheral blood (PBMC) were analysed for the presence of neoplastic B-cells by flow-cytometry (Fig. 2A–C). Disease infiltration was documented in the spleen also by immunohistochemical analysis where foci of neoplastic cell infiltrates, highlighted by human CD20^+^ immunoreactivity, were found in association with patient-derived T-cells, identified as human CD3^+^ cells^27,28^ (Fig. 2D,E).

**Figure 3:**
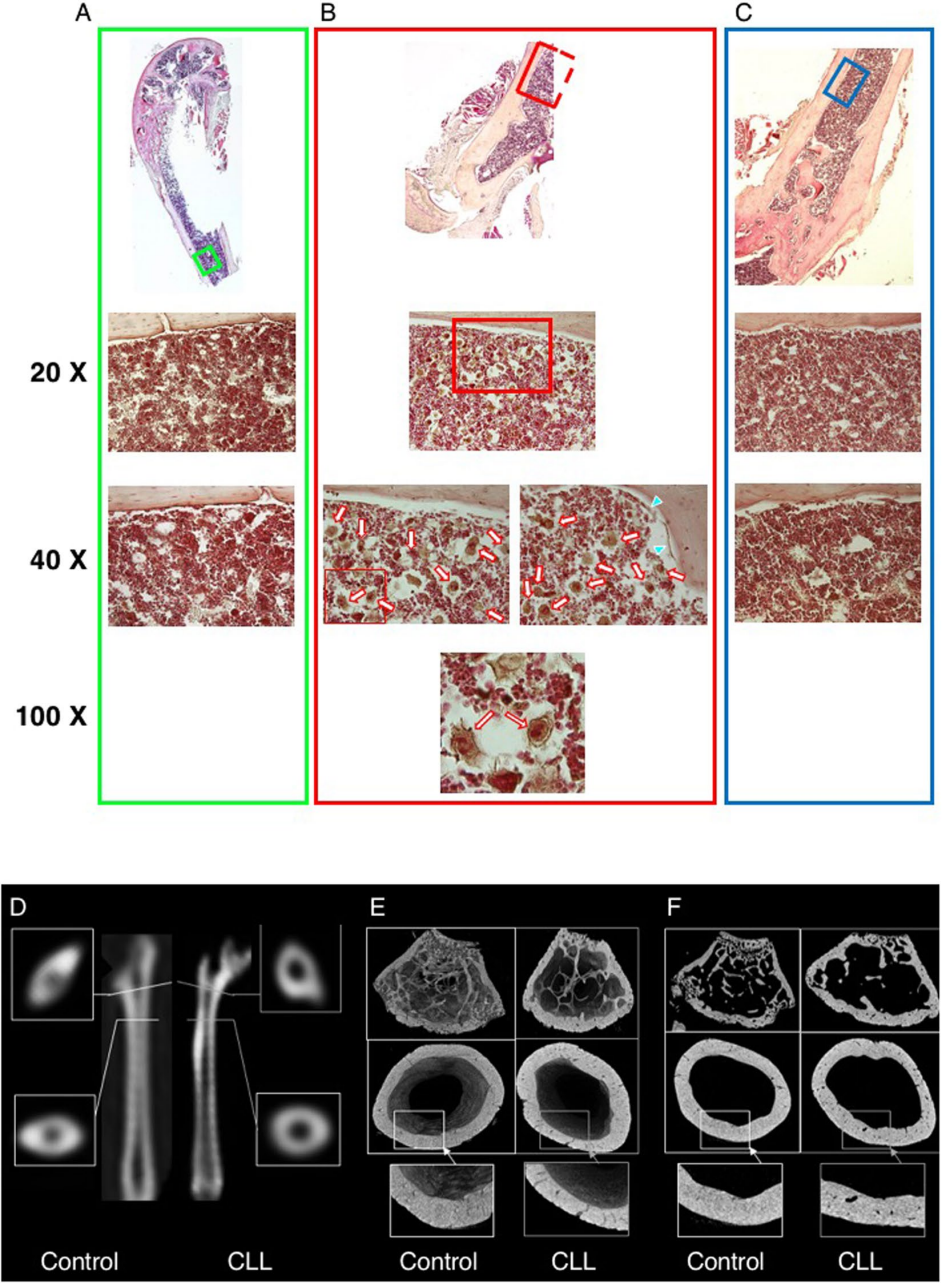
Panel A displays hematoxylin-eosin eosin staining of bone and bone marrow biopsy in a sham mouse with magnification related to the green insert. Panel B displays the same analysis in an untreated xenograft model (relative to the red insert): a high number of multinucleated large cells similar to osteoclasts are evident (white and red arrows). Panel C displays the absence of this finding in the bone marrow of a mouse subjected to Denosumab treatment for three weeks (blue insert). Panel D reports the original images of the right femur obtained by high resolution CT in a control model and in a CLL mouse, respectively. The longitudinal sections are represented in the middle and connected with the corresponding short axis slices in the original CT scans obtained in living animal. The enlargement in intrabone section is apparent. Alongside these images, Panel E displays a 3D representation of high resolution CT obtained *ex vivo* from the same femur. These images document a relative loss in trabecular structure as well as an irregular border of compact bone. This pattern is confirmed in the original 2D sections of the same microCT scan (panel F) in which compact bone erosion of femur shaft is detailed (bottom inserts).

Immunofluorescence analysis documented the presence of human cells in the BM of untreated models (Suppl. Figure 3) and also showed the presence of a high number of cells with osteoclast appearance. By contrast, this finding was not observed in control mice and, more importantly, in animals treated with Denosumab (Fig. 3A–C). Analysis of femur structure in xenograft models nicely confirmed the bone derangement observed in patients. In fact, the apparent bone erosion associated with CLL was evident *in vivo* at CT2 (Fig. 3D) and was visually confirmed *ex-vivo* by high-resolution CT images that provided a description of spatial distribution of bone tissue within the whole femur in 3D (Fig. 3E) and 2D reconstruction (Fig. 3F).

**Figure 4:**
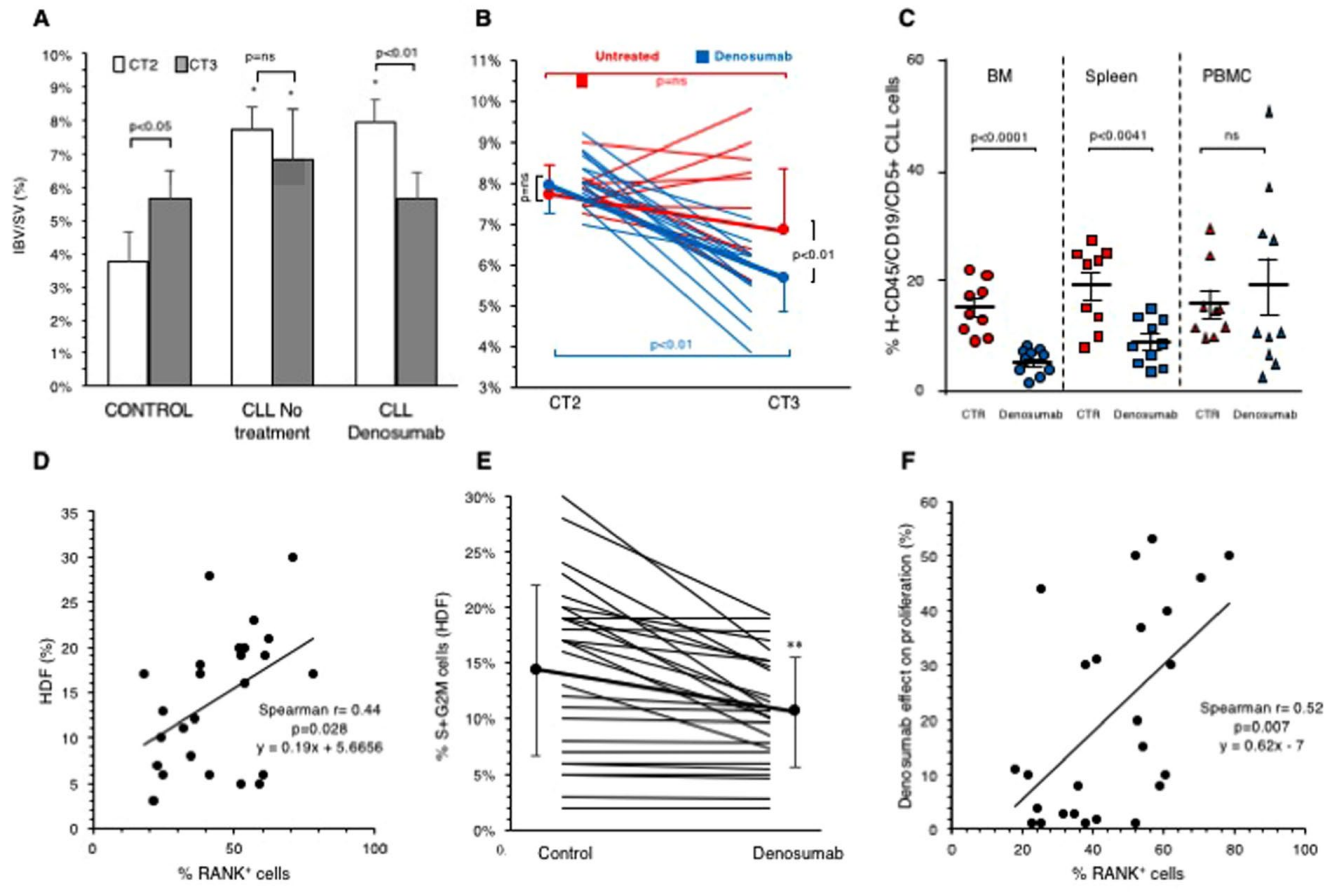
Panel A reports column chart of % IBV/SV ratio in control mice, CLL untreated mice (CLL no treatment) and CLL Denosumab ones. White columns indicate bone erosion at CT2 (four weeks after CLL cells administration) while grey columns report the same variable at CT3. AT CT2, CLL was associated with a significant femur erosion (*p < 0.01 vs controls). This difference persisted in untreated mice at CT3 (three weeks later). By contrast, Denosumab induced a significant reduction in IBV/SV to values that were lower with respect to untreated mice and similar to ones of control group. Panel B, displays the individual response to treatment (blue lines) or placebo (red lines) from CT2 to CT3. Denosumab caused a significant reduction of IBV/SV. Panel C summarizes the effect of in *vivo* denosumab treatment on human neoplastic B-cells (h CD45/CD5/CD19- positive cells). Percentage of CLL cells was significantly lower in denosumab treated mice compared to controls treated with saline (CTR) in BM and spleen. The mean and SEM of 19 mice injected with CLL cells from two different cases are shown (Mann-Whitney test). Panel D displays the direct correlation between %RANK^+^ B cells (X axis) in peripheral blood of each patient and actively proliferating cells indexed by HDF (Y axis). Panel E: displays the *in vitro* reduction in HDF induced by Denosumab. Panel F: displays the direct relationship between %RANK^+^ cells (X axis) and Denosumab effect on HDF.

Overall, CLL was associated with an increase in IBV/SV with respect to sham group at CT2 (7.2% ± 1.1% vs 4.2 ± 1.3%, respectively, p < 0.001) (Fig. 4A). In untreated CLL mice, femur IBV/SV ratio did not significantly change and remained higher than controls at CT3 (Fig. 4A). By contrast, Denosumab induced an apparent normalization of bone structure reducing IBV/SV ratio to values significantly lower with respect to untreated mice (Fig. 4B) and similar to the value reached by control group during the three weeks of aging (Fig. 4A).

The role of human CLL in bone erosion was further confirmed by the observation that, one week after the last treatment, number of neoplastic B-cells detected in BM and spleens was significantly higher in untreated xenograft models with respect to those treated with denosumab (Fig. 4C). Actually, no significant difference could be found in PBMC, in which a slight increase in peripheral neoplastic cells also occurred in some denosumab mice, probably due to mobilization from spleen and bone marrow lymphoid tissue after treatment. Nevertheless, the combined evaluation of bone histology, bone structure and CLL cells bulk indicates a possible correlation between RANK/RANKL pathway activation and bone erosion as well as in CLL cells survival and expansion in lymphoid tissue.

### Biological indicators of CLL – bone stroma interaction

Among the possible hormonal factors involved in bone erosion, osteopontin, osteoprotegerin, osteocalcin, parathyroid hormone and 1–25 OH vitamin D showed extremely variable levels and were not considered in the analysis (data not shown). Serum level of cytokines related to both CLL cell proliferation and osteoclast function, namely IL8 and TNFα^29,30^ were only partially related to disease severity. In fact, IL8 concentration was extremely variable and relatively independent from clinical risk (Suppl. Figure 4). By contrast (and in agreement with previous studies^31^), TNFα progressively increased throughout Binet stages (3.8 ± 2.67 pg/mL vs 11.5 ± 13.2 pg/mL and 13.5 ± 10.4 pg/mL in stage A, B and C, respectively, p < 0.05 at trend analysis) (Suppl. Figure 4).

Percentage of RANK^+^/RANKL^+^ cells was relatively stable in the different clinical stages. However, the increase in circulating clonal cells throughout Binet classes was associated with an increase in absolute number of RANK^+^/RANKL^+^ double positive (DP) leukemic cells (1677 ± 1990 cells/mmc vs 4337 ± 4768 cells/mmc and 10769 ± 12887 cells/mmc, in Binet A-B-C respectively, p < 0.01 at trend analysis, Suppl. Figure 4). A similar observation applied to total number of RANKL^+^ cells (3989 ± 3882 cells/mmc, vs 7639 ± 6291 cells/mmc and 19926 ± 19354 cells/mmc, in Binet A, B and C respectively, p < 0.01 at trend analysis, Suppl. Figure 4). By contrast, the total number of RANK^+^ cells increased only in Binet C stage (Suppl. Figure 4).

To further highlight the role of RANK-RANKL loop in promoting proliferation of the leukemic clone, the response of CLL cells to microenvironment-mimicking stimulation was tested *in vitro* under basal and RANK-RANKL-inhibited conditions. This study was performed in the 28/36 patients in whom peripheral blood sampling resulted in an adequate CLL cell number and included 10/16, 10/12 and 8/8 patients of stage A, B and C, respectively. Actively proliferating cells, measured as fraction of hyperdiploid cells (HDF), were significantly less represented in samples harvested from Binet A patients (8.6 ± 6%) with respect to both B and C stage (17.5 ± 5.6 and 17.6 ± 8.2%, p < 0.05 vs Binet A, respectively). HDF displayed a considerable level of positive correlation with %RANK^+^ cells (r = 0.44, p < 0.05, Fig. 4D) and, more importantly, was significantly reduced when the loop RANK-RANKL was impaired. In fact, Denosumab lowered HDF from 14.5 ± 7.4% to 10.7 ± 4.7% (p < 0.01, Fig. 4E), regardless of patient’s Binet stage. Moreover, Denosumab effect on cell proliferation – measured according to the calculation defined in the Methods – correlated with %RANK^+^ cells (r = 0.52, p = 0.007, Fig. 4F). Altogether, the *in vitro* results of this patient-by-patient analysis suggested the presence of a link between RANK-RANKL axis and the proliferating potential of each specific CLL clone.

**Figure 5:**
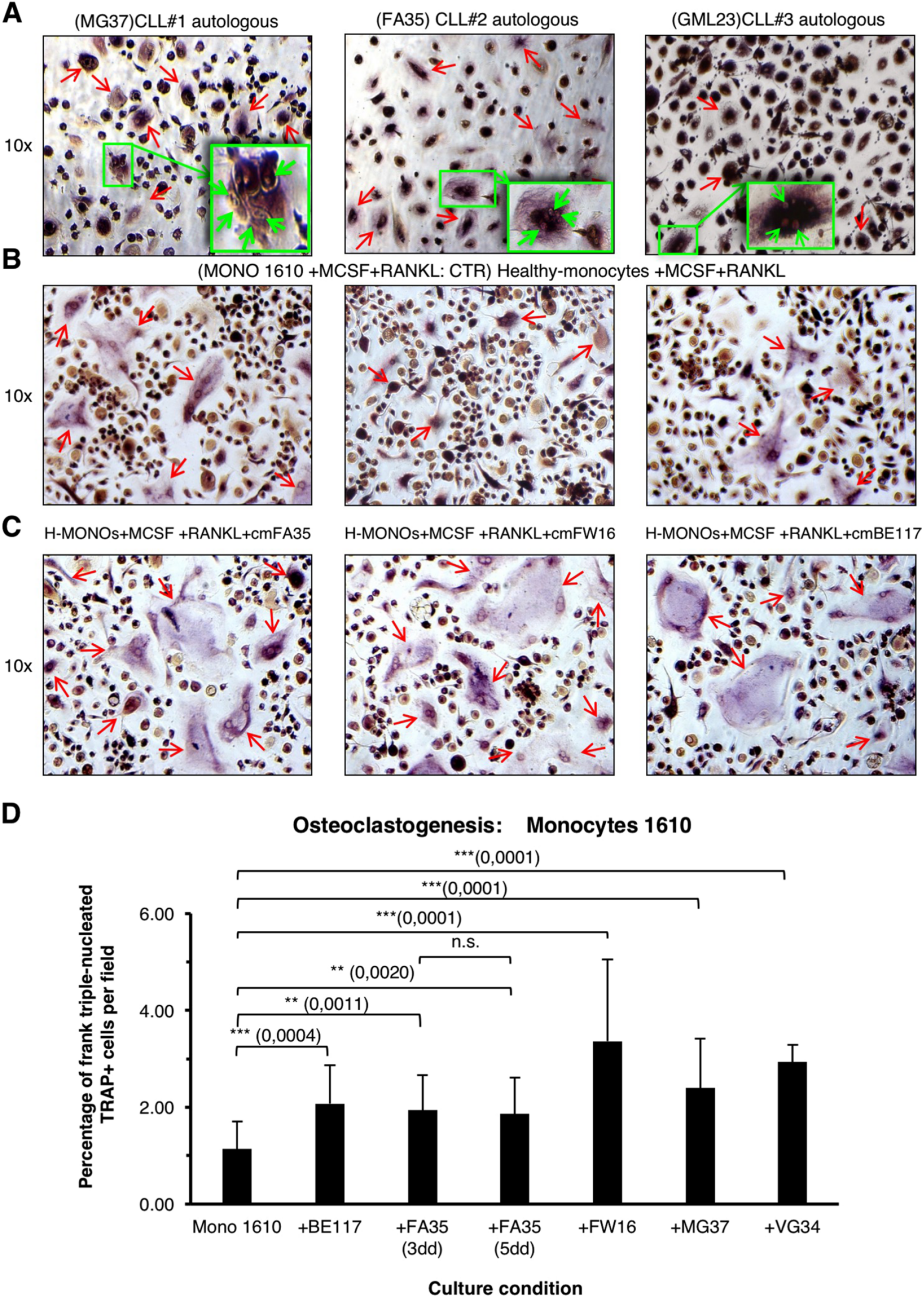
In panel A, cells with features of osteoclasts, as determined by TRAP positivity and ≥3 nuclei/cell (red arrows), were observed after co-cultures of monocytes with autologous CLL B cells for 25 days, without any monocytes pre-activation. Details at high magnification are displayed in the green box. In panel B, monocytes sampled from a healthy donor differentiated toward osteoclastic phenotype after activation with RANKL and MCSF. In panel C, number and the size of osteoclasts, from healthy monocytes pre-activated with MCSF + RANKL, appeared enhanced when conditioned media (CM) of CLL B cells cultures were added. Images are relative to experiments performed with CM from 3 representative CLL cases. In Panel D, histograms depict the average (±SD) percentage of tri-nucleated TRAP^+^ cells counted per field in all different experimental conditions: control CD14^+^ purified monocyte culture; +BE117, +FA35, +FW16, +MG37, +VG34: control monocytes cultured with the addition of the corresponding CM of CLL B cells. Nominal p values are indicated; n.s.: not significant. A minimum of total 3000 cells was counted for each culture condition and experiment.

We thus extended the evaluation of RANK-RANKL loop to verify its capability to promote osteoclastic commitment of circulating monocytes in CLL. As shown in Fig. 5A, cells with osteoclastic features (positive for TRAP and with 3 nuclei or more) were identifiable when autologous monocytes were cultured with CLL B cells for 25 days. Interestingly, this response occurred spontaneously, without prior MCSF and RANKL stimulation, which is the classic activation to obtain osteoclasts *in vitro*. These same images further allow to appreciate how different TRAP^+^ cells are fusing together giving rise to more typical large multinucleated osteoclasts.

In addition, we documented the capability of conditioned media from CLL B cells cultures to increase number and size of osteoclasts of healthy monocytes. In these cells, osteoclast commitment could be achieved only after activation with MCSF + RANKL (Fig. 5B). However, adding conditioned medium derived from CLL B cells cultures of 3 different patients, markedly increased osteoclast formation, as indicated by the increased percentages of the positive tri-nucleated TRAP^+^ cells in the culture wells (Fig. 5C).

### Prediction of skeletal erosion and BM metabolism

As shown in Table 2, univariate regression analysis documented a correlation between degree of compact bone erosion in the appendicular skeleton and disease aggressiveness as indicated by % CD38^+^ cells, TNFα and IL8 concentrations as well as BM FDG uptake. The role of RANK-RANKL loop became more evident at multivariate analysis in which the predictors of IBV/SV ratio were number of CLL RANKL^+^ cells, IL8 concentration and metabolic BM activation (N-SUV).

**Table 2:** Prediction of appendicular bone erosion: uni- and multivariate analysis.

	Univariate analysis					Multivariate analysis				
	Non standardized coefficient		Standardized coefficient			Non standardized coefficient		Standardized coefficient		
	B	Standard error	Beta	t	p	B	Standard error	Beta	t	p
RANK^+^ cell number	1.709 × 10^-7^	0.000	0.104	0.6	0.523	—	—	—	—	—
RANK^+^-RANKL^+^ cell number	8.806 × 10^−7^	0.000	0.192	1.124	0.269	—	—	—	—	—
% cells in S + G_2_M cycle phase	0.001	0.001	0.264	1.398	0.174	—	—	—	—	—
Hemoglobin	−0.041	0.026	−0.255	−1.538	0.133	—	—	—	—	—
**TNF-alpha**	**0.992**	**0.001**	**0.461**	**2.98**	**0.005**	—	—	—	—	—
CD19^+^ cells	1.464 × 10^−6^	0.000	0.18	1.068	0.293	—	—	—	—	—
**% CD38** ^**+**^ **cells**	**0.001**	**0.000**	**0.41**	**2.581**	**0.014**	—	—	—	—	—
**IL8**	**0.001**	**0.000**	**0.348**	**2.035**	**0.051**	**0.001**	**0.021**	**0.449**	**3.171**	**0.001**
**BM FDG uptake (N-SUV)**	**0.061**	**0.017**	**0.529**	**3.632**	**0.001**	**0.060**	**0.015**	**0.542**	**3.971**	**0.001**
**RANKL** ^**+**^ **cell number**	7.571 × 10^−7^	0.000	0.258	1.512	0.14	**0.000**	**0.000**	**0.376**	**2.62**	**0.016**

Determinants of FDG uptake within the intraosseous tissues are reported in Table 3. At univariate analysis, BM metabolism was strictly correlated with both TNFα levels and *in vitro* CLL cell proliferation, measured by HDF, while it was virtually independent from haemoglobin levels. However, only degree of appendicular compact bone erosion preserved the statistically significant correlation with RBM N-SUV at multivariate analysis.

**Table 3:** Prediction of FDG uptake in active bone marrow: uni- and multivariate analysis.

	Univariate analysis					Multivariate analysis				
	Non standardized coefficient		Standardized coefficient			Non standardized coefficient		Standardized coefficient		
	B	Standard error	Beta	t	p	B	Standard error	Beta	t	p
RANK^+^ cell number	7.37 × 10^−7^	0	0.052	0.299	0.767	—	—	—	—	—
RANK^+^-RANKL^+^ cell number	5.29 × 10^−6^	0	0.134	0.777	0.443	—	—	—	—	—
**% cells in S + G**_**2**_ **M cycle phase**	**0.031**	**0.008**	**0.557**	**3.86**	**0.001**	—	—	—	—	—
Hemoglobin	−0.003	0.002	−0.227	1.257	0.184	—	—	—	—	—
**TNF-alpha**	**0.015**	**0.004**	**0.507**	**3.383**	**0.002**	—	—	—	—	—
CD19^+^ cells	1.431 × 10^−6^	0	0.204	1.215	0.233	—	—	—	—	—
% CD38^+^ cells	0.003	0.002	0.205	1.223	0.23	—	—	—	—	—
IL8	0.001	0.003	0.067	0.366	0.717	—	—	—	—	—
RANKL^+^ cell number	5.32 × 10^−6^	0	0.211	1.223	0.23	—	—	—	—	—
**Appendicular skeleton IBV/SV**	**4.56E + 00**	**1.257**	**0.529**	**3.632**	**0.001**	**5.507**	**1.471**	**0.607**	**3.744**	**0.001**

## Discussion

The major finding of the present study is that CLL is associated with significant bone erosion in all clinical stages. This skeletal alteration was particularly evident in long bone shafts, was documented in all risk classes, progressively increasing from Binet A to Binet C, and was related to the number of CLL RANKL^+^ cells. Also, the same long bone shafts were colonized by metabolically active RBM, as documented by PET-FDG imaging. The spatial coherence of RBM activity and bone erosion matches the capability of human CLL cells to reproduce skeletal derangement in immune-deficient mice and to promote osteoclast commitment in autologous and healthy monocytes. Altogether, these observations indicate the presence of an interaction between neoplastic clone and bone microenvironment at least partially involving RANK-RANKL loop.

CLL was associated with an array of skeletal alterations that cannot be attributed to the confounding interference of previous therapies, since it occurred in treatment-naïve patients. Several studies on experimental models of leukemia and myeloma already reported an alteration of trabecular bone structure and composition in vertebral bodies^32,33^. Similarly, our computational approach documented an expansion of IBV at the expense of a cortical thinning. This erosion was particularly evident in appendicular structures of our patients while it did not reach the statistical significance in axial skeletal segments regardless disease stage. This partial disagreement might probably reflect the limited spatial resolution of the low-dose attenuation-correction CT scanning protocol and the consequent influence of the partial volume averaging effect in bones characterized by a thin cortical layer^22–24^. This methodological limitation does not permit to ascertain whether CLL-associated bone erosion involves specific bone segments or rather the whole skeleton. However, this same feature confirms the robustness of the signal whose documentation does not require dedicated scanning procedures and high radiation burden.

Actually, shaft cortical thinning progressively increased with increasing clinical index of disease severity. This evidence at least partially agreed with the distribution of RBM and its correlation with Binet staging as documented by FDG uptake. Usually, RBM metabolism cannot by detected by FDG imaging in long bone shafts of adult humans, coherently with the concept that, in adulthood, hematopoietic activity is limited to the intraosseous space of axial skeleton and the proximal epiphyses^34–36^. The present data do not permit to ascertain whether this colonization can be ascribed to the presence of the neoplastic clone or rather the expansion of normal hematopoietic tissue. Nevertheless, previous studies already reported a link between disease aggressiveness and BM metabolic activity detected at PET CT^37–39^. Moreover, the observed distribution pattern did not reproduce the usual response to myelotoxic damage or increased need of circulating blood cells^40,41^ that implies an activation of RBM in axial skeleton. Accordingly, the match between cortical thinning and BM metabolic activation in the long bone shafts may suggest an interaction between CLL clone and bone microenvironment. This hypothesis is supported by the analysis of xenograft models in which human CLL cells expanded IBV in mouse femurs. Similarly, it is confirmed by the *in vitro* evidence: CLL-monocytes displayed a spontaneous commitment toward large and multinucleated cells. Although some large cells may represent “nurse-like” CLL cells^42–44^, a substantial number of osteoclasts are clearly identifiable based on the presence of 3 or more nuclei together with TRAP positivity^44^.

Altogether, these findings indicate that CLL cells may disrupt the delicate osteoclastic/osteoblastic balance eventually altering the physiological bone remodelling.

Regarding the mechanisms underlying this interaction, several studies already documented an aberrant RANKL expression in different B cell malignancies such as CLL, multiple myeloma and follicular lymphoma^13–21^. Actually, Secchiero *et al*. reported that RANK/RANKL interaction potently increases the release of IL-8 as a factor able to promote clone proliferation and resistance to apoptotic signals in CLL^14^. Similarly, Borge *et al*. described a high production of soluble RANKL by leukemic cells in a CLL patient with bone destruction^45^.

In the present study, the role of RANK/RANKL loop was confirmed by several observations. In xenograft models, CLL administration was associated with a thinning of femoral cortex. Due to the nature of xenograft model and the role of activated T cells in CLL engraftment^27^, the present data do not permit to verify whether this phenomenon was selectively caused by the neoplastic clone or by the inflammatory condition favored by the co-administered T lymphocytes^46^ even in the absence of an overt xenograft versus host reaction (X-GVHD). Nevertheless, antagonizing human RANKL with Denosumab markedly reduced the number of osteoclasts visible at histology, diminished femur bone erosion and restored the ratio between trabecular and compact bone volumes close to the control value documenting the role of RANK/RANKL axis and its capability to activate osteoclast commitment in circulating monocytes^20,21^. On the other hand, *in vitro* proliferating activity of stimulated CLL clone was directly correlated with the prevalence of RANK + cells and was significantly reduced by the same Denosumab. Accordingly, the present data suggest that RANK/RANKL loop activation – promoted by either CLL clone or autologous T lymphocytes – might extend its role to represent a factor enhancing CLL proliferating potential beyond its recognized anti-apoptotic effect.

Finally, the role of this loop was confirmed *in vivo* by the uni- and multivariate analyses that indicated RANKL^+^ cell number as a predictor of bone erosion together with RBM metabolic activity, % of CD38^+^ CLL cells and serum levels of both TNFα and IL8.

In conclusion, the present study confirms the presence of a bone erosion in a small prospectively enrolled population of consecutive, treatment-naïve CLL patients. This skeletal derangement is present in all disease stages, it becomes more evident with increasing clinical risk index and particularly involves long bone shafts at least partially recolonized by active BM. This finding indicates that the interaction between leukemic clone and microenvironment might play a significant role in the natural history of CLL. This interaction at least partially involves RANK-RANKL pathway whose activation, if confirmed, may represent a possible therapeutic target. Similarly, this same relationship can suggest that bone erosion (if properly confirmed in larger populations studies) could represent a surrogate end-point for testing the efficacy of new treatments.

## Methods

### Patient Recruitment

Enrolments included 36 consecutive previously untreated CLL patients (11 females, mean age 70 ± 12 years; range 43–91 years). CLL diagnosis had been established made from peripheral blood cells immunophenotype and staging was performed according to Binet classification^26^. All patients had not previously received therapy or had been free from therapy for at least 6 months. Exclusion criteria included history or concomitant presence of other solid or hematologic neoplasm, previous prolonged corticosteroid therapy, uncontrolled diabetes and active or chronic infection as well as autoimmune disease. Thirty-six age- and sex-matched healthy subjects, randomly selected from a published normalcy database, served as controls^22^. The study was approved by the Ethics Committee of IRCCS San Martino/IST Hospital. All patients signed an informed consent prior to study inclusion. All experiments were performed in accordance with relevant guidelines and regulations.

### PET/CT acquisition and reconstruction

All patients underwent ^18^F-fluorodeoxyglucose (FDG) PET/CT imaging after a minimum of 12 hours of fasting. Before the study, height and weight were measured, ideal body weight (IBW) was calculated according to Robinson formulation^47^ and serum glucose level was assessed ( ≤ 1.5 g/L). FDG (4.8–5.2 MBq per kilogram of body weight) was intravenously administered and, after minimum 60–75 of resting in the waiting room, scanning was performed in 3D mode from vertex to toes in an arms-down position, using an integrated PET/CT scanner (Hirez; Siemens Medical Solutions, Knoxville, US). PET raw data were reconstructed by means of ordered subset expectation maximization (OSEM, three iterations, 16 subsets) and attenuation correction was performed using CT data. The transaxial field of view and pixel size of the reconstructed PET images were 58.5 cm and 4.57 mm, respectively, with a 128 × 128 matrix. A 16–detector row helical CT scan was performed with non-diagnostic current and voltage settings, with a gantry rotation speed of 0.5 second and a table speed of 24 mm per gantry rotation. The entire CT data set was fused with the three-dimensional PET images by using an integrated software interface (Syngo; Siemens, Erlangen, Germany).

### Image analysis

Image analysis was performed according to the previously validated method^22–24^. In brief, the application sequentially identifies and quantifies compact (CBV) and trabecular bone volume (IBV) in all transaxial CT images, using the appropriate attenuation characteristics of these two tissues. Skeletal erosion was assessed by measuring the ratio between trabecular and total skeletal volume (IBV/SV, where SV is the sum of IBV and CBV). The computational analysis was first carried out on the whole skeleton; then segmental analysis was performed on axial skeleton (vertebral bodies and sternum) and appendicular long bone shafts. All volumetric data were normalized according to ideal body weight (IBW). FDG uptake within IBV was thus analysed to estimate the volume of red BM (RBM) defined by a standardized uptake value (SUV) ≥ 1.11^22^. Finally, overall metabolic activity of RBM was corrected for blood-pool tracer concentration to obtain a normalized metabolic index (N-SUV).

### Analysis of Biomarkers and Soluble Mediators

A 50-ml venous blood example was obtained before injection of the tracer. CLL cells were isolated from patients’ peripheral blood by means of Ficoll-Hypaque (Seromed, Biochrom) density gradient centrifugation. Clonal cell number was estimated by the measurement of CD5/CD19/CD23 triple positive B-cells that was determined by direct immunofluorescence with monoclonal antibodies (mABs) to CD19-FITC (BD Biosciences Pharmigen, San Josè California, USA), CD23-PE (BD Biosciences), and CD5-PC5 (Beckman Coulter Immunotech, Marseille, France). CD38 expression status was tested using flow cytometry analysis by triple staining with CD19 FITC (BD Biosciences), CD38 PE (BD Biosciences), and CD5 PC5 (Beckman Coulter) mABs. CLL clones were defined as CD38^+^ when CD38 was expressed on > 30% of the leukemic clone^48^. *IGHV* mutational status was determined as previously described^49^.

Expression of both RANK and RANKL on cellular surface was assessed by means of flow-cytometry analysis using a combination of monoclonal antibody anti-human CD19-FITC (BD Biosciences), RANK-PE (R&D System, clone#80704), CD5-PE-vio770 (Milteny), RANKL-APC (Biolegend, clone MIH24). Presence of these markers on clonal elements permitted to estimate the fraction of CLL cells positive for RANK/RANKL (double positive, DP), RANK^+^ and RANKL^+^ cells. Each fraction was multiplied by the absolute number of CD5^+^CD19^+^ to obtain the total number of cells/mmc. Flow cytometry analysis was performed by FACSCanto (BD Biosciences) and DIVA 6 software (BD Biosciences).

In the same blood sample, serum levels of TNFα and IL-8 were measured using Millipore’s Milliplex MAP Human Bone Magnetic Bead Kit and acquired by MAGPIX instrumentation (#HBNMAG-51K and HCYTOMAG-60K).

### Cultural Analysis

*In vitro* cultures were set up by co-culturing CLL cells and CD40L^+^ murine fibroblasts in the presence of IL-4. Inhibition of RANK-RANKL loop was achieved by the addition of the human monoclonal anti-RANKL antibody Denosumab (Xgeva, Amgen) to stimulated CLL cultures at a concentration of 105 micrograms/ml (730 nM). Cell vitality was measured by flow cytometry (FACS Calibur, Becton Dickinson, San Jose, CA, USA) through propidium iodide exclusion assays and defined as vital cell percentage after 96 hours in unstimulated cultures. Cell proliferation was measured by flow cytometric DNA content histograms as previously described^50^ and defined by the % of cells in S + G_2_M cell cycle phases (hyper diploid fraction, HDF) after a five-days activation. Response to Denosumab was defined by the formula:

1 $$Denosumab\,Response=\frac{HD{F}_{Control}-HD{F}_{Denosumab}}{HD{F}_{Control}}$$

### Experimental reproduction of skeletal erosion pattern caused by CLL

To evaluate the interaction between CLL and bone stroma, 20, six to eight-week-old female NOD-SCID IL-2R γ-chain null (NSG) mice (The Jackson Laboratory), a xenograft model for CLL growth *in vivo* ^27,28^, were housed in sterile enclosures under specific pathogen-free conditions. All mice were intravenously inoculated with 8 × 10^7^ peripheral blood cells sampled from two CLL patients (n = six each, respectively) while 10 further mice served as control group. All procedures involving animals were performed in respect of the current National and International regulations and were reviewed and approved by the Licensing and Animal Welfare Body of the IRCCS-AOU San Martino-IST National Cancer Research Institute, Genoa, Italy. Hind limb high-resolution CT scans (GE Lightspeed pro32) were performed at week #2, #6 and #9 post-xenograft according to the following parameters (40 mA, 140 kV, DFOV 9. 6 cm, SFOV 32 cm, thickness of slices 0.625 mm). After confirming a successful engraftment at TC2, CLL mice was divided into two subgroups: The first subgroup (n = 10) was treated with Denosumab (5 mg/Kg once a week for three weeks consecutively), while the remaining (n = 10) did not receive any treatment. Reoriented slices of both femurs were analyzed with the same algorithm used for human skeleton analysis to estimate SV, IBV and CBV. After the sacrifice, the right femur was isolated in the last animal of both xenograft and control groups to be scanned using a high-resolution micro-TC (Bruker SkyScan 1272) at a spatial resolution of 4 µm in all three spatial dimensions.

In order to exclude infection by human CMV quantitative determination of CMV DNA was performed by real-time PCR on frozen mice plasma (pulled for groups of treatment) or serum patients’ using the CMV HHV-6, 7, 8 amplification Kit (Argene, bioMérieux,Varilhes, France) as recommended by the manufacturer’s instructions. Extraction and amplification steps were performed by DNA extraction Kit (Argene) and QUIA AMP DNA Blood mini KIT (Quiagen), (Argene). Samples were run in Rotor Gene 3000 instrument.

### Bone histology and immunofluorescence

Lower limbs of each animal were dissected, cleared of skin and other soft tissues, washed in phosphate-buffered saline (PBS) and fixed in paraformaldehyde (4% in PBS) for two days at 4 °C. Untill further processing, specimens were maintained in 70% Ethanol. Subsequently specimens were rinsed in PBS and decalcified in Osteodec (Bio-Optica S.p.A., Milan, Italy; cat. N. 05-MO3005) for two days, changing the decalcifying solution every 12 hrs, at RT, and dehydrated by subsequent washings in 70%, 90%, 95% and 100% Ethanol (two hours each). After one overnight in Cedar oil (Merck KGaA, Darmstad, Germany; cat. N. 106965), specimens were included in paraffin, with standard procedures. Sample murine femurs were then cut orthogonally to the bone main axis, separating epiphyses from diaphysis, and cut again in halves. Standard re-inclusion procedures were performed to obtain final sections for subsequent stainings.

Sections (10 μm thickness) were de-waxed and re-hydrated with standard procedures and finally washed in PBS for 15 min. Sections were stained with Meyer’s hematoxylin (Bio-Optica S.p.A., Milan, Italy; cat. N. 05-MO6002) for 5 minutes, rinsed under tap water for 5 minutes and stained again with a freshly prepared Eosin-Erythrosine solution (0.2% in water; Eosin and Erythrosine: Merck KGaA, Darmstad, Germany; cat. N. 115935 and N. 115936, respectively). Subsequent dehydration was performed in ethanol (95%, 100%, three times for 5 minutes each) and ultimately in xilol. Stained sections were mounted with Eukitt hardening-mounting medium (Bio-Optica S.p.A., Milan, Italy; cat. N. 09–00500).

For immunostaining, de-waxed sections were re-hydrated, washed in PBS, permeabilized for 10 minutes at RT (permeabilization solution: 0.1% Triton X-100; 0.1% Na-citrate, in PBS) and pre-incubated with goat anti-serum for 30 min at RT to block aspecific sites. After a brief wash in TBS buffer (20 mM Tris/HCl; 0.5 M NaCl; 0.1% Tween20; bovine serum albumin 3%), sections were incubated overnight at 4 °C with an anti-human mitochondria antibody (Acris GmbH, Germany; cat. N. BM608; 1:200 in TBS). Sections were then washed (3 times for 5 minutes each) and challenged with a goat anti-mouse Fluorescein (FITC)-conjugated secondary antibody (Jackson Research Labs. Inc.; West Groove, PA, USA; cat. N. 115-095-146) for 3 hrs at 4 °C.

Images were acquired with a Nikon Digital Sight DS-5Mc camera, mounted on an Olympus BX5 1 fluorescence microscope, using Nikon imaging software NIS-Elements F, release 2.20.

### Differentiation of Osteoclasts from CLL-monocytes co-cultured with leukemic B cells or from allogeneic monocytes of healthy donors cultured with conditioned media from CLL B cells cultures

To define whether CLL B cells may affect differentiation of autologous monocytes toward osteoclasts we cultured PBMCs from CLL patients at 2 × 106 /ml/well in 24 well plates. Attached cells were gently washed with Phosphate Buffered Saline (D-PBS; Dulbecco’s Phosphate Buffered Saline; EuroClone, cat. n. ECB4004L) and subsequently stained for Tartrate Resistant Acid Phosphatase (TRAP) by the use of a commercial kit (Leukocyte Acid Phosphatase (TRAP) Kit; Sigma cat. N. 387A-1Kit), following manufacturer’s instructions. The presence and the number of osteoclast-like TRAP^+^ multinucleated (3 or more) cells was determined visually on an optical inverted microscopy (Olympus CKX41) equipped with a Nikon Digital Sight DS-U1 camera and the Nikon NIS-Element F 2.0 imaging software. Experiments were performed in duplicate and at least 3 images for each well were acquired. Images were then scored for tri-nucleated TRAP^+^ cells, counting both positive and negative cells. A minimum of 3000 total cells was counted for each culture condition, in each experiment. To determine whether the addition of medium derived from CLL B cells cultures (1 × 10^6^ CLL B cells × 72 h) could increase the formation of osteoclasts from monocytes purified from healthy donors, CD14^+^ monocytes were purified from PBMCs of healthy donors by the use of CD14-conjugated magnetic beads following manufacturer’s instructions. (Milteny). 1 × 10^5^ cells/well of purified CD14^+^ cells were thus seeded in a 24 well plate with RPMI1640 + FCS10%. After one day, recombinant human MCSF (25 ng/ml) and RANKL (25 ng/ml) were added to these cultures and after 3 more days each well was refeeded with new culture medium additioned of MCSF + RANKL. After further 3 days, conditioned media (CM) from CLL B cells cultures were added together with a suboptimal concentration of MCSF and RANKL and after 7 days more the plates were examined for the presence of TRAP^+^ multinucleated cells, as described above. Each experiment was performed in duplicate.

### Statistical Analysis

All data were reported as means of ± standard deviation or proportions, as appropriate. Unpaired or paired t tests were used to compare data in different groups or in the same group, as appropriate. Univariate and Multivariate Linear regression analyses were performed by using the least squares method. A p value of less than 0.05 was considered statistically significant. All statistical analyses were carried out by using a dedicated software package (SPSS, version 20; SPSS, Chicago, Ill).

## Electronic supplementary material

Supplementary figures

## References

[1] Howlader, N. *et al*. SEER Cancer Statistics Review, 1975–2014, National Cancer Institute. https://seer.cancer.gov/csr/1975_2014/results_single/sect_13_table.07.pdf (2017).

[2] Zent CS, Kyasa MJ, Evans R, Schichman SA (2001). Chronic lymphocytic leukemia incidence is substantially higher than estimated from tumor registry data. Cancer..

[3] Dores GM (2007). Chronic lymphocytic leukaemia and small lymphocytic lymphoma: overview of the descriptive epidemiology. Br J Haematol..

[4] Abrisqueta P (2009). Improving survival in patients with chronic lymphocytic leukemia (1980–2008): the Hospital Clinic of Barcelona experience. Blood..

[5] Pulte D (2016). GEKID Cancer Survival Working Group. Trends in survival of chronic lymphocytic leukemia patients in Germany and the USA in the first decade of the twenty-first century. J Hematol Oncol..

[6] Podhorecka M (2016). Deregulation of apoptosis - is it still an important issue in pathogenesis of chronic lymphocytic leukemia?. Curr Cancer Drug Targets..

[7] Messmer BT (2005). *In vivo* measurements document the dynamic cellular kinetics of chronic lymphocytic leukemia B cells. J Clin Invest..

[8] Damle RN, Calissano C, Chiorazzi N (2010). Chronic lymphocytic leukaemia: a disease of activated monoclonal B cells. Best Pract. Res. Clin. Haematol..

[9] Rossi D, Gaidano G (2016). Richter syndrome: pathogenesis and management. Semin Oncol..

[10] Lazarian G (2016). TP53 mutations are early events in chronic lymphocytic leukemia disease progression and precede evolution to complex karyotypes. Int J Cancer..

[11] Gentile M (2014). Prospective validation of a risk score based on biological markers for predicting progression free survival in Binet stage A chronic lymphocytic leukemia patients: results of the multicenter O-CLL1-GISL study. Am J Hematol..

[12] Gribben JG (2010). How I treat CLL up front. Blood..

[13] Barcala V (2003). RANKL expression in a case of follicular lymphoma. Eur J Haematol..

[14] Secchiero P (2006). Role of the RANKL/RANK system in the induction of interleukin-8 (IL-8) in B chronic lymphocytic leukemia (B-CLL) cells. J Cell Physiol..

[15] Wong BR, Josien R, Choi Y (1999). TRANCE is a TNF family member that regulates dendritic cell and osteoclast function. J Leukoc Biol..

[16] Schmiedel BJ (2013). RANKL expression, function, and therapeutic targeting in multiple myeloma and chronic lymphocytic leukemia. Cancer Res..

[17] Xu ZS (2015). Constitutive activation of NF-κB signaling by NOTCH1 mutations in chronic lymphocytic leukemia. Oncol Rep..

[18] Gasparini C, Celeghini C, Monasta L, Zauli G (2014). NF-κB pathways in hematological malignancies. Cell Mol Life Sci..

[19] Kong YY, Boyle WJ, Penninger JM (1999). Osteoprotegerin ligand: a common link between osteoclastogenesis, lymph node formation and lymphocyte development. Immunol Cell Biol..

[20] Hsu H (1999). Tumor necrosis factor receptor family member RANK mediates osteoclast differentiation and activation induced by osteoprotegerin ligand. Proc Natl Acad Sci USA..

[21] Terpos E (2009). RANKL inhibition: clinical implications for the management of patients with multiple myeloma and solid tumors with bone metastases. Expert Opin Biol Ther..

[22] Sambuceti G (2012). Estimating the whole bone-marrow asset in humans by a computational approach to integrated PET/CT imaging. Eur J Nucl Med Mol Imaging..

[23] Fiz F (2015). Allogeneic cell transplant expands bone marrow distribution by colonizing previously abandoned areas: an FDG PET/CT analysis. Blood..

[24] Fiz F (2014). Adult advanced chronic lymphocytic leukemia: computational analysis of whole-body CT documents a bone structure alteration. Radiology..

[25] Langenberg, J. C., Bosman, W. M., Van den Bremer, J. & Ritchie, E. D. Pathological fractures in a patient with chronic lymphatic leukaemia without disease progression. *BMJ Case Rep*. **25**, 10.1136/bcr-2014-208118 (2015). 10.1136/bcr-2014-208118 PMC4342641 25716040

[26] Hallek M (2008). Guidelines for the diagnosis and treatment of chronic lymphocytic leukemia: a report from the InternationalWorkshop on Chronic Lymphocytic Leukemia updating the National Cancer Institute–Working Group 1996 guidelines. Blood..

[27] Bagnara D (2011). A novel adoptive transfer model of chronic lymphocytic leukemia suggests a key role for T lymphocytes in the disease. Blood..

[28] Cutrona, G. *et al*. Effects of miRNA-15 and miRNA-16 expression replacement in chronic lymphocytic leukemia: implication for therapy. Leukemia. 2017 Feb 3. doi: 10.1038/leu.2016.394. [Epub ahead of print] 10.1038/leu.2016.394 28053325

[29] Wierda WG (2003). Plasma interleukin 8 level predicts for survival in chronic lymphocytic leukaemia. Br J Haematol..

[30] Binsky I (2007). IL-8 secreted in a macrophage migration-inhibitory factor- and CD74-dependent manner regulates B cell chronic lymphocytic leukemia survival. Proc Natl Acad Sci USA..

[31] Ferrajoli A (2002). The clinical significance of tumor necrosis factor-alpha plasma level in patients having chronic lymphocytic leukemia. Blood..

[32] Frisch BJ (2012). Functional inhibition of osteoblastic cells in an *in vivo* mouse model of myeloid leukemia. Blood..

[33] Horger, M. *et al*. Improved MDCT monitoring of pelvic myeloma bone disease through the use of a novel longitudinal bone subtraction post-processing algorithm. *Eur Radiol*. **23**, 10.1007/s00330-016-4642-6 (2016). 10.1007/s00330-016-4642-6 27882427

[34] Hayman JA (2011). Distribution of proliferating bone marrow in adult cancer patients determined using FLT-PET imaging. Int J Radiat Oncol Biol Phys..

[35] Cristy M (1981). Active bone marrow distribution as a function of age in humans. Phys Med Biol..

[36] Vande Berg BC, Malghem J, Lecouvet FE, Maldague B (1998). Magnetic resonance imaging of the normal bone marrow. Skeletal Radiol..

[37] Bruzzi JF (2006). Detection of Richter’s transformation of chronic lymphocytic leukemia by PET/CT. J Nucl Med..

[38] Mauro FR (2015). Diagnostic and prognostic role of PET/CT in patients with chronic lymphocytic leukemia and progressive disease. Leukemia..

[39] Shaikh F, Janjua A, Van Gestel F, Ahmad A (2017). Richter Transformation of Chronic LymphocyticLeukemia: A Review of Fluorodeoxyglucose Positron Emission Tomography-Computed Tomography and Molecular Diagnostics. Cureus..

[40] Rose BS (2012). Correlation between radiation dose to ^18^F-FDG-PET defined active bone marrow subregions and acute hematologic toxicity in cervical cancer patients treated with chemoradiotherapy. Int J Radiat Oncol Biol Phys..

[41] Piva R (2014). ^18^F-fluorodeoxyglucose PET/CT in aplastic anemia: a literature review and the potential of a computational approach. Clin. Pract..

[42] Burger JA (2000). Blood-derived nurse-like cells protect chronic lymphocytic leukemia B cells from spontaneous apoptosis through stromal cell-derived factor-1. Blood..

[43] Tsukada N (2002). Distinctive features of “nurselike” cells that differentiate in the context of chronic lymphocytic leukemia. Blood..

[44] Wekerle H, Ketelsen EP, Ernst M (1980). Thymic nurse cells. J Exp Med..

[45] Borge M (2016). Soluble RANKL production by leukemic cells in a case of chronic lymphocytic leukemia with bone destruction. Leuk. Lymphoma..

[46] D’Amico L, Roato I (2012). Cross-talk between T cells and osteoclasts in bone resorption. Bone KEy Reports..

[47] Robinson JD, Lupkiewicz SM, Palenik L, Lopez LM, Ariet M (1983). Determination of ideal body weight for drug dosage calculations. Am J Hosp Pharm..

[48] Damle RN (1999). Ig V gene mutation status and CD38 expression as novel prognostic indicators in chronic lymphocytic leukemia. Blood..

[49] Fais F (1998). Chronic lymphocytic leukemia B cells express restricted sets of mutated and unmutated antigen receptors. J Clin Invest..

[50] Bruno S (2015). Metformin inhibits cell cycle progression of B-cell chronic lymphocytic leukemia cells. Oncotarget..

