# Supplementary material for: Functional Activation of Osteoclast Commitment in Chronic Lymphocytic Leukaemia: a Possible Role for RANK/RANKL Pathway
Source: Sci Rep. 2017 Oct 26;7:14159. doi: 10.1038/s41598-017-12761-1 (PMC5658396; doi:10.1038/s41598-017-12761-1)
Supplement: Supplementary file 1 — Supplementary figures [file 41598_2017_12761_MOESM1_ESM.pdf]

Functional Activation of Osteoclast Commitment in Chronic Lymphocytic Leukaemia: a Possible Role for RANK/RANKL Pathway.

Cecilia Marini[1,2,3,*], Silvia Bruno[4], Francesco Fiz[3,5], Cristina Campi[6], Roberta Piva[3], Giovanna Cutrona[7], Serena Matis[7], Alberto Nieri[3], Maurizio Miglino[8], Adalberto Ibatici[9], Anna Maria Orengo[2], Anna Maria Massone[6], Carlo Emanuele Neumaier[10], Daniela de Totero[11], Paolo Giannoni[12], Matteo Bauckneht[3], Michele Pennone[2], Claudya Tenca[4], Elena Gugiatti[4], Alessandro Bellini[3], Anna Borra[3], Elisabetta Tedone[9], Hülya Efetürk[2,3], Francesca Rosa[10], Laura Emionite[13], Michele Cilli[13], Davide Bagnara[4,14], Valerio Brucato[15], Paolo Bruzzi[16], Michele Piana[6,17], Franco Fais[4,7], Gianmario Sambuceti[2,3].

[1] CNR Institute of Bioimages and Molecular Physiology, Milan, Italy.
[2]Nuclear Medicine IRCCS AOU San Martino-IST, Genoa, Italy
[3]Nuclear Medicine, Department of Health Science, University of Genova, Genoa, Italy.
[4]Department of Experimental Medicine, University of Genoa, Genoa, Italy.
[5]Nuclear Medicine Unit, Department of Radiology, Uni-Klinikum Tuebingen, Germany.
[6]SPIN Institute, CNR, Genoa, Italy.
[7]Molecular Pathology, IRCCS AOU San Martino-IST, Genoa, Italy.
[8]Department of Internal Medicine, University of Genoa, Genoa, Italy.
[9]Haematology Clinic, IRCCS AOU San Martino-IST, Genoa, Italy.
[10]Radiology Unit, IRCCS AOU San Martino-IST, Genoa, Italy.
[11]Transfer Gene Laboratory, IRCCS AOU San Martino-IST, Genoa, Italy.
[12]Stem Cell Laboratory, Department of Experimental Medicine, University of Genova, Genoa, Italy
[13]Animal Facility, IRCCS AOU San Martino-IST, Genoa, Italy.
[14]Karches Center for Oncology Research, Feinstein Institute for Medical Research, Northwell Health, Manhasset, NY, USA
[15]Department of Civil, Environmental, Aerospace, Materials Engineering (DICAM), Aten Center, CHAB pole, University of Palermo, Italy.
[16]Epidemiology and Clinical trial Service, IRCCS AOU San Martino-IST, Genoa, Italy.
[17]Department of Mathematics (DIMA), University of Genoa, Genoa, Italy.

***Corresponding author:**

Dr. Cecilia Marini
C/o Nuclear Medicine Unit
Department of Health Sciences
Pad. Sommariva, IRCCS San Martino – IST
Largo Rosanna Benzi 10, 16132 Genoa, Italy
E-Mail : Cecilia.marini@unige.it
Secondary E-Mail: C1570@unige.it

Total word count (manuscript): 4945

Word Count (Abstract): 190

Disclosures: The authors declare no competing financial interests.

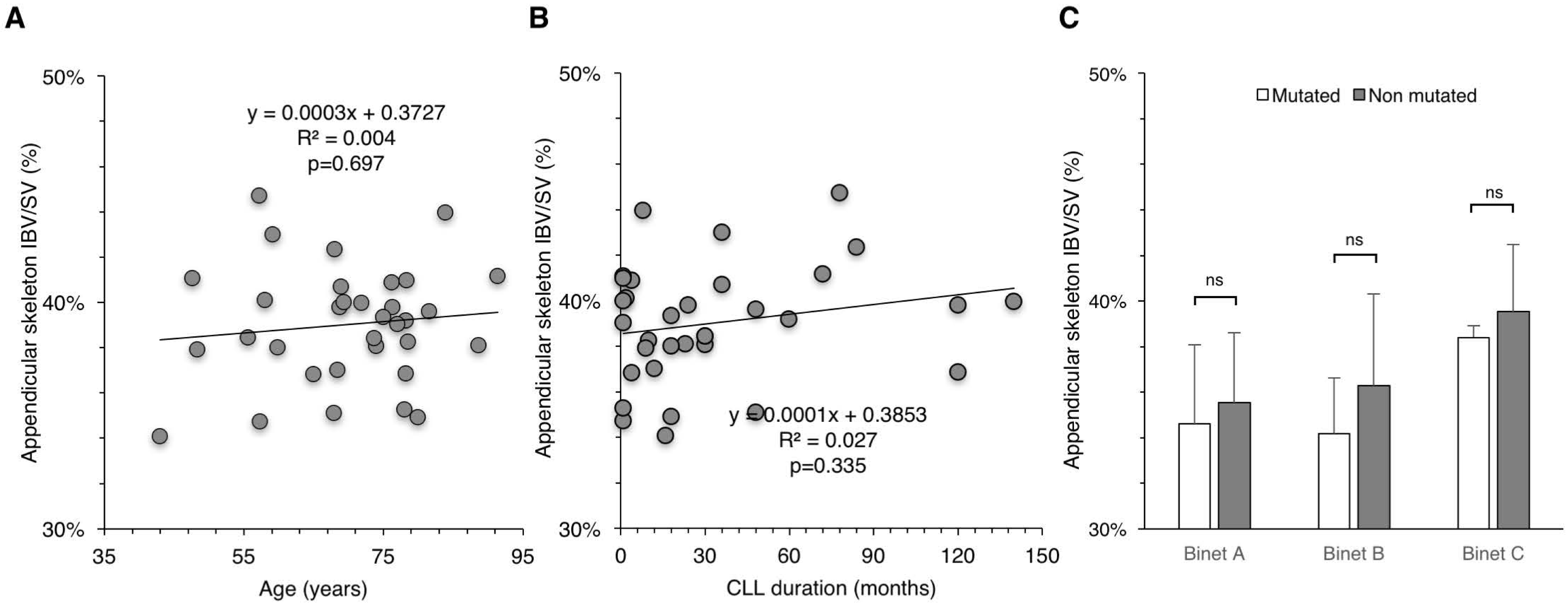

Lack of correlation between IBV/SV ratio in the appendicular skeleton (Y axis) and patient age (panel A), time gap between disease diagnosis and study date (panel B). Similarly, as displayed in panel C, <2% cells with IgHV mutation (grey columns, non mutated) did not identify a higher bone erosion with respect to low risk pattern.

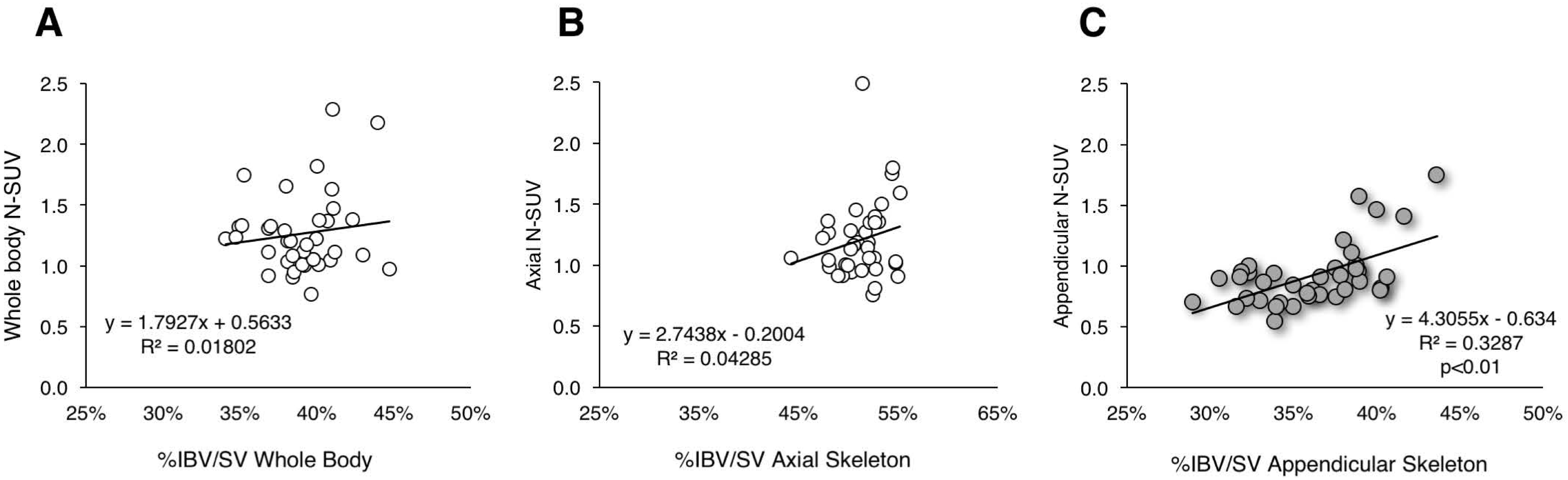

The metabolic activation of RBM was not related to bone erosion, estimated by IBV/SV ratio, when the whole (panel A) or axial skeleton (Panel B) were analysed. By contrast, these two variables were strictly correlated within the appendicular bones (Panel C).

Supplementary Figure #3

CLL-treated (FW 1)

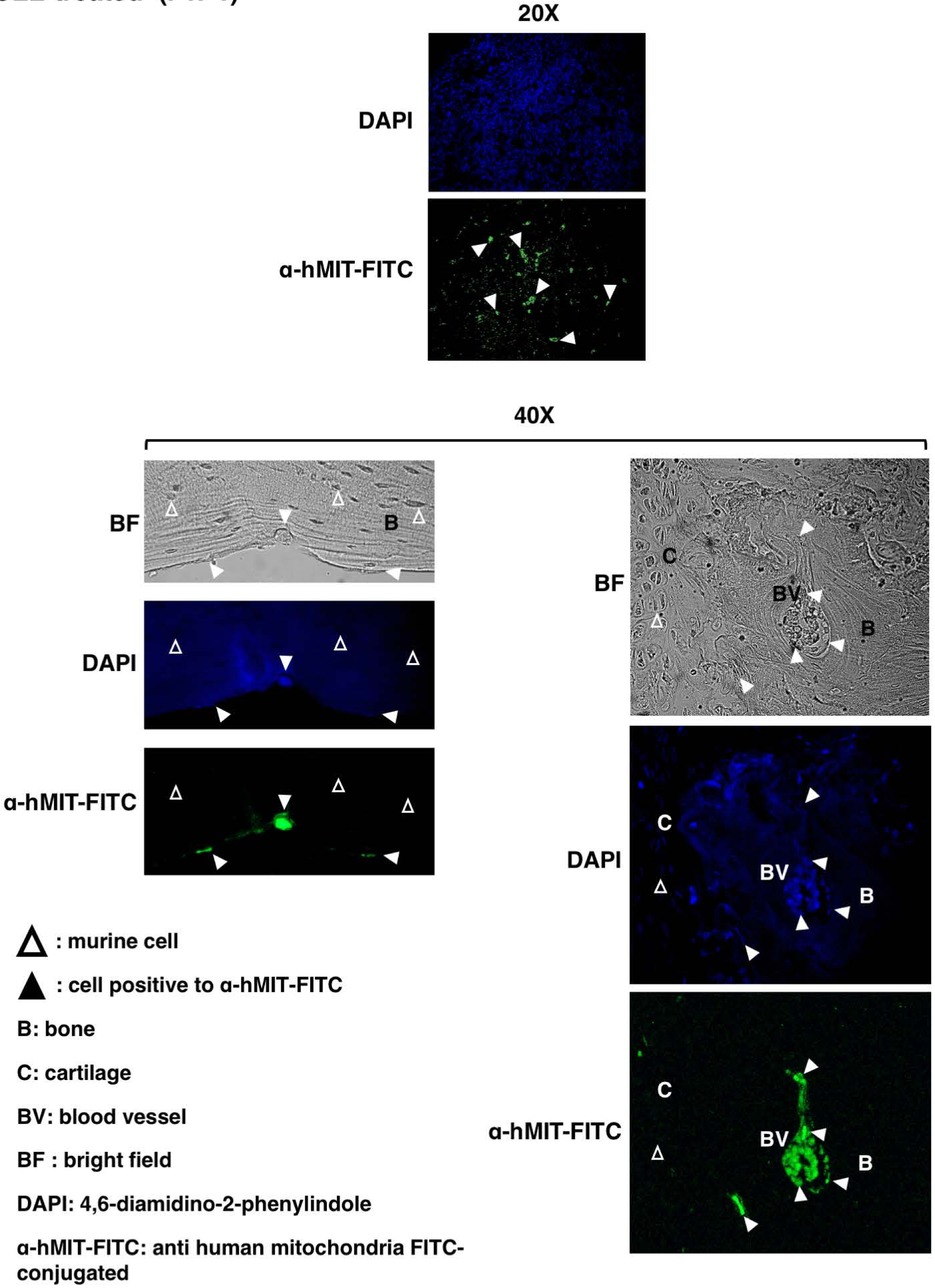

Supplementary Figure 3: Immuno-fluorescence images of a xenograft CLL untreated mouse. At small magnification, imaging with4,6-diamidino-2-phenylindole (DAPI) and α-hMIT-FITC: anti human mitochondria FITC-conjugated documents the presence of human mitochondria among nucleated cells in mouse bone marrow. This localization is confirmed a higher magnification (40x) in which mouse and human cells are identified.

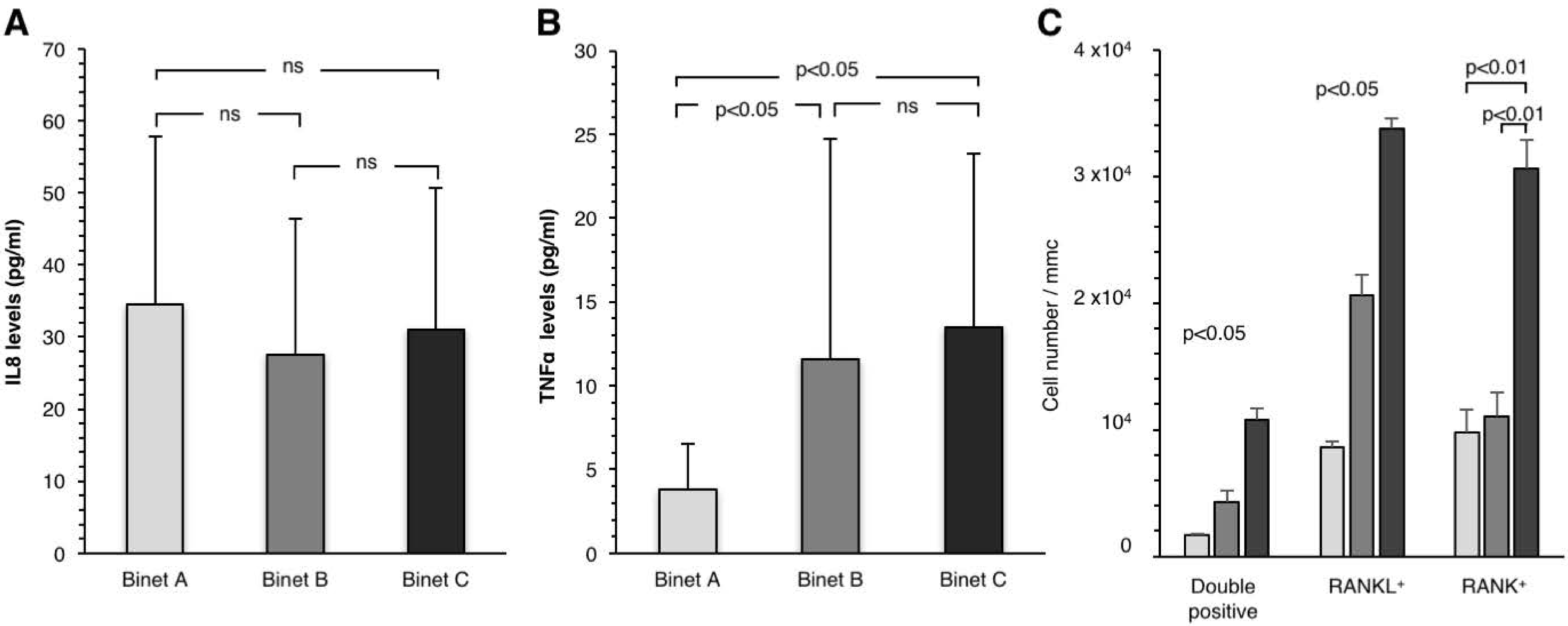

Serum levels of IL8 (panel A) and TNFα (panel B) in Binet A, B and C patients (means ± standard deviation). Panel C displays means and SEM values of total number of peripheral blood RANK^{+}, RANKL^{+} and double positive (DP) leukemic cells/mmc, reported separately for Binet A (pale grey), B (grey) or C (dark grey) patients;
